# **Forensic DNA Applications: An Interdisciplinary Perspective** – **a new book in forensic science**

**DOI:** 10.3325/cmj.2014.55.434

**Published:** 2014-08

**Authors:** Dragan Primorac, Moses Schanfield

**Affiliations:** 1 University of Split, School of Medicine, Split, Croatia; 2 “J. J. Strossmayer” University, School of Medicine, Osijek, Croatia; 3 “Sv. Katarina” Hospital, Zabok, Croatia; 4 Penn State University, Eberly College of Science, State College, PA, USA; 5 University of New Haven, West Haven, CT, USA; 6 George Washington University at MVC, Washington, DC, USA

To the Editor:

Forensic DNA Applications: An Interdisciplinary Perspective (2014; Edited by Dragan Primorac and Moses Schanfield. CRC Press, Taylor Francis Group. 23 Chapters, 621 pages) is intended for different types of audiences interested in forensic medicine. It could be both a textbook for forensic molecular biology students and a reference book for practitioners of forensic molecular biology. Moreover it is expected to be used by lawyers and judges dealing with civil and criminal cases involving DNA technology. The book covers the ever-growing field of forensic DNA testing, and an effort has been made to present state of the art knowledge on each topic at the time of submission (March 2013).

The book was developed as an outgrowth of the Sixth ISABS Conference on Human Genome Project Based Applications in Forensic Science, Anthropology and Individualized Medicine, bi-annual educational conference of the International Society of Applied Biological Sciences (ISABS) held in Split, Croatia, June 1-5, 2009. It should also be noted that *the Croatian Medical Journal* (CMJ) is the official publication of ISABS and thus far ISABS has published 9 thematic issues of the *CMJ*, each issue related to one of the conferences. It was felt at these conferences that there was a need for a new book that would be used as a textbook but also as a reference book for people working in the field of forensic molecular biology as well as individuals investigating and adjudicating cases involving DNA evidence, regardless if they are civil or criminal cases. The approach is international, and therefore the information given could not be considered relevant to analysts only working in the US or Europe. Moreover as the title states the approach is “interdisciplinary.” The majority of authors are speakers and participants at the ISABS bi-annual meeting (1). The next meeting will take place on June 22-26, 2015 in Bol, island of Brač, Croatia. This will be a new opportunity to present to the *CMJ* new submissions related to the leading trends in forensic science.

In the book, there are many texts on forensic molecular biology, some authored by a single author, others by a team of experts. This particular volume is unique in the sense that the 51 authors that provided the 23 chapters are experts in their particular areas, meaning that they work in the areas that they are writing about (Table 1-4). This edition has 4 sections and 23 chapters, representing all aspects of forensic DNA methodology, ethics, law, and policy. This book encompasses the period before DNA testing was done through the present and therefore can provide a unique perspective on the history and practice of forensic DNA testing. The authors come from Australia, Bosnia and Herzegovina, Croatia, the Czech Republic, Finland, the Netherlands, Slovenia, and the United State of America. This book is important because it brings together all of the current areas of interest and research in a single up-to-date volume (2).

**Table 1:** **SECTION 1. GENERAL BACKGROUND AND METHODOLOGICAL CONCEPTS** (Mitchell M. Holland, Theresa Caragine, Section Editors)

**1.Basic Genetics and Human Genetic Variation**	*Dragan Primorac, Moses S. Schanfield and Damir Marjanović*	Chapter 1 is a review of forensic DNA testing including screening methods from the beginning of RFLP testing through current STR, SNP testing and sequencing.
**2. Forensic DNA Analysis and Statistics**	*Moses S. Schanfield, Dragan Primorac and Damir Marjanović*	Chapter 2 covers forensic DNA analysis emphasizing the statistical aspects of nuclear, X, Y and mtDNA testing.
**3. Forensic aspects of mtDNA analysis**	*Mitchell* *M. Holland and Gordan Lauc*	Chapter 3 is a review of marker testing on mtDNA including hypervariable regions I and II and the SNP markers in the coding region.
**4. Y Chromosome in Forensic Science**	*Manfred Kayser, Kaye N. Ballantyne*	Chapter 4 is a review of Y SNPs and STRs and their role in the definition of haplogroups and haplotypes and the calculation of statistics.
**5. Forensic Application of X Chromosome STRs**	*Toni Marie Diegoli*	Chapter 5 looks at the new world of X chromosome STRs and the problems in calculating statistics in a haplodiploid system. This is one of the first reviews of X chromosome markers.
**6. Low Copy Number DNA profiling**	*Theresa Caragine, Krista Currie, and Craig O’ Connor*	Chapter 6 is a review of testing for DNA when components are in too small an amount or degraded, either because of being in a mixture or sample condition. This is an exhaustive treatment of testing strategy and statistics from the first laboratory in the USA that validated LCN testing.
**7. Forensic DNA Mixtures, Approaches, and Analysis**	*Theresa Caragine, Adele Mitchell and Craig O’Connor*	Chapter 7 is the logical extension of the LCN chapter (Chapter 6) and covers the various approaches to the interpretation and calculation of statistics in forensic cases with mixtures.
**8. Forensic DNA Typing and Quality Assurance**	*Daniel Vanek and Katja Drobnič*	Chapter 8 covers quality assurance and accreditation under the US ASCLD-LABORATORY and ISO 17025 standards.

**Table 4:** **SECTION 4. LAW, ETHICS AND POLICY** (Fredrick Bieber, David H. Kaye, Section Editors)

**20. DNA as Evidence in the Courtroom**	*David H. Kaye, Frederick R. Bieber, and Damir Primorac*	Chapter 20 is about the use of DNA evidence in the courtroom, and provides some insight to both the European and US systems of jurisprudence.
**21. Some Ethical Issues in Forensic Genetics**	*Erin D. Williams, and David H. Kaye*	Chapter 21 covers the general concepts of bioethics, and then goes on to deal with ethical issues in acquiring DNA samples, DNA databanks, phenotyping and ancestry identification, identification of remains, as well as, the ethics of report writing and testimony.
**22. DNA in Immigration and Human Trafficking**	*Sara Huston Katsanis and Joyce Kim*	Chapter 22 delves into the area of DNA testing in immigration cases and human trafficking. The chapter also deals with the ethical, legal and social considerations of this type of DNA identification testing.
**23.DNA Databases**	*Christopher Asplen*	Chapter 23 deals with the international perspectives on forensic DNA databases. This chapter is up to date and inclusive of data on a worldwide basis, including US Supreme Court decisions in June of 2013 from a global perspective.

## 

**Table 2:** **SECTION 2. USES AND APPLICATIONS** (Timothy Palmbach, Jenifer Smith, Section Editors)

**9. Collection and Preservation of Physical Evidence**	*Henry C. Lee, Timothy M. Palmbach, Dragan Primorac, and Šimun Anđelinović,*	Chapter 9 covers an international approach to the collection and preservation of biological evidence though the title says physical evidence.
**10. Identification of Missing Persons and Mass Disaster Victim identification by DNA**	*Barbara A. Butcher, Frederick R. Bieber, Zoran M. Budimlija, Sheila M. Dennis, and Mark A. Desire*	Chapter 10 concentrates on the seemingly unending task of identifying remains from single or mass casualty events, whether it is a skeleton found in a park, the World Trade Center 9/11 event or the recent war in Croatia and Bosnia and Herzegovina.
**11. Bioterrorism and Forensic microbiology**	*Alemka Markotić, James W. Le Duc, and Jennifer Smith*	Chapter 11 provides a history and modern approach to the identification of microbiological select agents. This area has not been covered previously in books of this type.
**12. Forensic Animal DNA Analysis**	*Marilyn Menotti-Raymond*, *Victor A. David, Stephen J. O’Brien, Sree Kanthaswamy, Petar Projić, Vedrana Škaro, Gordan Lauc, and Adrian Linacre*	Chapter 12 is an extensive review of many of the areas involving animal genetics in forensics from our common pets (dogs and cats) to farm animals to the protection of wild animals. Another chapter not normally covered in reviews of forensic DNA.
**13. Application of DNA-Based Methods in Forensic Entomology**	*Henry D. Wells and Vedrana Škaro*	Chapter 13 explores the two sides of forensic entomology: Identifying the species of insects from maggots and identifying the human source from the DNA in the maggot’s gut. Another original chapter for this type of publication.
**14.Forensic Botany**: **Plants as Evidence in Criminal Cases and Agents of Bioterrorism**	*Heather Miller Coyle, Henry C. Lee, and Timothy M. Palmbach*	Chapter 14 goes into the multitude of area of forensic botany, from the identification of clones of marijuana plants to the use of pollen to trace movements of a body, to the plant bioterrorism agents such as ricin.

**Table 3:** **SECTION 3. RECENT DEVELOPMENTS AND FUTURE DIRECTIONS IN HUMAN FORENSIC MOLECULAR BIOLOGY** (Manfred Kayser and Antti Sajantila, Section Editors)

**15. Forensic Tissue Identification with Nucleic Acids**	*Dmitrty Zubakov and Manfred Kayser*	Chapter 15 reviews the classical methods for tissue identification and the newer RNA based techniques to identify tissues, including tissues such as menstrual blood that could not be identified previously.
**16. Evolving Technologies in Forensic DNA Analysis**	*Cassandra D. Calloway and Henry Erlich*	Chapter 16 reviews some of none sequencing based technology to look at mtDNA and the advances in Next Generation Sequencing (NGS) and their applications to forensic science.
**17. Prediction of Physical Characteristics, such as Eye, Hair and Skin Color Based Solely on DNA**	*Elisa Wurmbach*	Chapter 17 is on the use of DNA SNP technology to predict eye, hair and skin color as an investigational tool in forensic DNA testing. Another new subject previously only covered in articles.
**18. Molecular Autopsy**	*Grace Axler-DiPerte, Frederick R. Bieber, Zoran M. Budimlija, Antti Sajantila, Donald Siegel, and Yingying Tang*	Chapter 18 has two major sections, the genomics of sudden natural death, and the use of toxicogenetics / pharmacogenetics in cause of death investigations. An area of increasing interest as the role of the genes that metabolize drugs and the risk to patients is studied in greater detail.
**19. Genetic Genealogy in the Genomic Era**	*Jake K. Byrnes, Natalie M. Myers, and Peter A. Underhill*	Chapter 19 has limited application to forensic DNA analysis. However, this area is getting a lot of attention in the popular media, and provides useful background information in this area of public excitement though the information is limited to broad areas of ancestry.
